# LncRNA-AL035458.2/hsa-miR-181a-5p Axis-Mediated High Expression of NCAPG2 Correlates With Tumor Immune Infiltration and Non-Small Cell Lung Cancer Progression

**DOI:** 10.3389/fonc.2022.910437

**Published:** 2022-05-19

**Authors:** Xi Chen, Jishu Guo, Wenjun Ren, Fan Zhou, Xiaoqun Niu, Xiulin Jiang

**Affiliations:** ^1^ Department of Neurosurgery, The Second Affiliated Hospital of Kunming Medical University, Kunming, China; ^2^ Institute for Ecological Research and Pollution Control of Plateau Lakes, School of Ecology and Environmental Science, Yunnan University, Kunming, China; ^3^ Department of Respiratory Medicine, The 2nd Affiliated Hospital of Kunming Medical University, Kunming, China; ^4^ Department of Cardiovascular Surgery, The First People's Hospital of Yunnan Province, Kunming, China; ^5^ Hematology and Rheumatology Department, The Pu’er People’s Hospital, Pu’er, China; ^6^ Department of Respiratory Medicine, Second Hospital of Kunming Medical University, Kunming, China; ^7^ Key Laboratory of Animal Models and Human Disease Mechanisms of Chinese Academy of Sciences & Yunnan Province, Kunming Institute of Zoology, Kunming, China; ^8^ Kunming College of Life Science, University of Chinese Academy of Sciences, Beijing, China

**Keywords:** NCAPG2, non-small cell lung cancer, prognosis biomarker, cell proliferation, cell migration, ceRNA

## Abstract

Lung adenocarcinoma (LUAD) is the most common histological lung cancer, and it is the leading cause of cancer-related deaths worldwide. NCAPG2 (non-SMC condensin II complex subunit G2) has been shown to be upregulated in various human cancers. Nevertheless, the underlying biological function and potential mechanisms of NCAPG2 driving the progression of LUAD remain unclear. In this study, we investigated the role of NCAPG2 in LUAD and found that the expression of NCAPG2 in LUAD tissues was significantly higher than that of NCAPG2 expression in adjacent normal tissues. Kaplan–Meier survival analysis showed that patients with higher NCAPG2 expression correlated with unfavorable clinical outcomes. Receiver operating characteristic (ROC) curve analysis showed that the AUC value of NCAPG2 was 0.914. Correlation analysis showed that NCAPG2 expression was associated with immune infiltration in LUAD. Finally, we found that AL139385.1 was upregulated in LUAD cancer tissues and cell lines. Knockdown of NCAPG2 inhibited cell proliferation, cell migration, and cell invasion of LUAD *in vitro*. More importantly, we established the AL035458.2/hsa-miR-181a-5p axis as the most likely upstream ncRNA-related pathway of NCAPG2 in LUAD. In conclusion, our data demonstrated that ncRNA-mediated high expression of NCAPG2 was correlated with progression and immune infiltration, and could serve as a prognostic biomarker for LUAD.

## Introduction

Lung cancer is one of the most predominant cancers in terms of morbidity and mortality worldwide (1). Due to the high rate of under-diagnosis of early-stage lung cancer, the disease is already in a progressive or advanced stage by the time it is diagnosed, and the 5-year survival rate in patients is under 20% (2). Lung cancer can be divided into small cell lung cancer and non-small cell lung cancer (NSCLC) depending on its histopathological features (3). In addition, lung adenocarcinomas (LUADs) are the largest histological subtype of NSCLC and account for approximately 40% of it (4). Recently, great progress has been made in the treatment of lung cancer. However, the easy distant metastasis, poor prognosis, and frequent recurrence still make LUAD a refractory disease. Thus, it is urgent to study the molecular mechanism underlying LUAD progression and seek effective therapeutic targets.

NCAPG2 (non-SMC condensin II complex subunit G2) is well characterized for its roles in cell mitosis. It has been shown that NCAPG2 plays an essential role in chromosome condensation and segregation during mitosis (5). Previous studies have indicated that NCAPG2 was increased in glioblastoma tissues and was correlated with poor clinical outcomes. Knockdown of NCAPG2 inhibited cell proliferation, migration, and invasion and modulated cell cycle in GMB (6). Meanwhile, NCAPG2 overexpression has also been found in hepatocellular carcinoma, and its higher expression was associated with adverse clinical outcomes. Forced NCAPG2 expression promotes cell proliferation, migration, and invasion by activating STAT3 and NF-κB signaling pathways in HCC (7). Recently, it was demonstrated that NCAPG2 may be a new therapeutic target and biomarker for future treatment and prognosis in colon cancer (8). In our previous study, we developed a new method called CVAA (Cross-Value Association Analysis), which functions without a normalization and distribution assumption. We applied it to large-scale pan-cancer transcriptome data generated by The Cancer Genome Atlas (TCGA) project and successfully discovered numerous new differentially expressed genes (DEGs) (9). NCAPG2 is one of these DEGs. However, the expression levels, clinical significance, biological function, and underlying mechanism of NCAPG2 in LUAD have not been reported.

In this study, we utilized TCGA, GTEX, HPA, and UALCAN datasets, and Kaplan–Meier (KM) plotter web to analyze NCAPG2 expression and its association with expression and clinical significance. Meanwhile, the correlation between NCAPG2 expression and immune infiltration was analyzed to explore the potential mechanisms involved in NCAPG2 modulation in the progression of LUAD. Finally, the biological function of NCAPG2 was identified in LUAD. Through a series of correlation, expression, and survival analyses, we assessed noncoding RNAs (ncRNAs) that contribute to higher expression of NCAPG2 in LUAD. We established the AL035458.2/hsa-miR-181a-5p axis as the most likely upstream ncRNA-related pathway of NCAPG2 in LUAD. In summary, our study confirmed the potential role of NCAPG2 in regulating tumor progression and its potential application in the diagnosis and prognostic evaluation of LUAD.

## Materials and Methods

### Data Processing and Differential Expression, Survival, and Correlation Analysis

We obtained raw counts of RNA-sequencing data and corresponding clinical information of tumor tissues and adjacent tissues from 33 types of cancer *via* the TCGA dataset and Genotype-Tissue Expression (GTEX) databases. In LUAD, we downloaded data in lung cancer tissues and lung normal tissues. All analytical methods were carried out using R software version v3.6.3. Expression analysis and KM survival curves were drawn with “ggplot2”, “survminer”, and “survival” R packages. Log-rank tests were used to determine significance, and univariate Cox proportional hazards regression was used to estimate *p*-values and hazard ratio (HR) with 95% confidence interval (CI) in KM curves. Two-gene correlation analysis was drawn with the R package “ggstatsplot”. The correlation between quantitative variables was assessed using Pearson’s correlation or Spearman’s correlation analysis.

### UALCAN Database

UALCAN (http://ualcan.path.uab.edu/) is an online resource for analyses of TCGA gene expression data (10). In this finding, we used UALCAN to determine the protein level of NCAPG2 in LUAD.

### Kaplan–Meier Plotter Analysis

The KM plotter (http://kmplot.com/analysis/), acting as an online database (11), is used to explore the influence of multiple genes on the prognosis of 21 different types of cancers. We used the KM plotter to explore the prognostic values of miRNAs in LUAD.

### PrognoScan Database Analysis

The correlation between NCAPG2 expression and overall survival (OS) in lung cancer was also examined by the PrognoScan database (http://www.abren.net/PrognoScan/) (12).

### The Human Protein Atlas

HPA (https://proteinatlas.org/) includes the normal tissue and tumor tissue protein levels of human gene expression profile information (13). In this study, we explored the protein expression of NCAPG2 in lung cancer tissue.

### ENCORI Database Analysis

The Encyclopedia of RNA Interactomes (ENCORI) database is a database for discovering the connection between miRNA–ncRNA and miRNA–mRNA (14). We used ENCORI to predict upstream potential miRNAs and lncRNAs that interact with NCAPG2 and hsa-miR-181a-5p. Meanwhile, we utilized ENCORI database to determine the correlation between lncRNA, miRNAs, and NCAPG2 in lung cancer.

### Gene Set Enrichment Analysis

In the present research, we utilized the LinkedOmics database (http://www.linkedomics.org/login.php) obtained from the co-expression genes of NCAPG2 in LUAD. We used GSEA software and the ClusterProfiler package to perform KEGG enrichment analysis of the signaling pathway of NCAPG2 in LUAD (15–17). We used the R package “ClusterProfiler” to perform Gene ontology (GO) enrichment and Kyoto Encyclopedia of Genes and Genomes (KEGG) pathway analyses of co-expression genes, visualized by the R package “ggplot2”.

### Cell Culture

The BEAS-2B cell line was purchased from the Cell Bank of Kunming Institute of Zoology, and cultured in BEGM media (Lonza, CC-3170). Lung cancer cell lines, including A549, H1299 and H1650, were purchased from Cobioer, China with STR documents, and were cultured in RPMI-1640 medium (Corning) supplemented with 10% fetal bovine serum (FBS) and 1% penicillin/streptomycin.

### Cell Proliferation and Cell Migration Assay

Cell proliferation assay was performed as previously described (9). Indicated tumor cells were plated onto 12-well plates, and the cell numbers were subsequently counted each day using an automatic cell analyzer countstar (Shanghai Ruiyu Biotech Co.). For the trans-well migration assay, 2×10^4^ cells/well in 100 μl of serum-free medium were plated in a 24-well plate chamber insert, and the lower chamber was filled with 10% FBS. After incubation for 24 h, cells were fixed with 4% PFA, washed, and then stained with 0.5% crystal violet to further capture pictures.

### The Clinical Proteomic Tumor Analysis Consortium Common Data Analysis

The Clinical Proteomic Tumor Analysis Consortium (CPTAC) has produced large proteomics datasets from the mass spectrometric interrogation of tumor samples previously analyzed by the TCGA program (18). In this study, we determined the expression of DTYMK in lung cancer using CPTAC data.

### Real-Time RT-PCR Assay

In the real-time RT-PCR assay, cells were lysed by RNAiso Plus (Takara Bio, Beijing, China, Cat. 108-95-2). Total RNAs were extracted according to the manufacturer’s protocol, and then reverse transcribed by using the RT reagent kit (Takara Bio, Beijing). The primers used in this study are as follows: β-actin-F: AAGTGTGACGTGGACATCCGC, β-actin-R: CCGGACTCGTCATACTCCTGCT, NCAPG2-F: TACAAGCCGTGTCTAAGGAGC, NCAPG2-R: TTGAGCCATGTTCGGTTTCCA.

### Immunohistochemical Staining

For immunohistochemical staining, the sections were deparaffinized in xylene and rehydrated through graded ethanol. Antigen retrieval was performed for 20 min at 95°C with sodium citrate buffer (pH 6.0). After quenching endogenous peroxidase activity with 3% H_2_O_2_ and blocking non-specific binding with 1% bovine serum albumin buffer, sections were incubated overnight at 4°C with the indicated primary antibodies. Following several washes, the sections were treated with HRP-conjugated secondary antibody for 40 min at room temperature, and stained with 3,3-diaminobenzidine tetrahydrochloride (DAB). The photographs were analyzed based on the ratio of the staining with the Image-Pro Plus 7.0 software.

### Statistical Analyses

All statistical analyses were performed using R (V 3.6.3), and ROC curves were used to detect NCAPG2 cutoff values using pROC packages. For the data regarding the function of NCAPG2, GraphPad Prism 7.0 was used for statistical analyses. The significance of the data between two experimental groups was determined by Student’s *t*-test, and multiple group comparisons were analyzed by one-way ANOVA. *p* < 0.05 (*), *p* < 0.01 (**), and *p* < 0.001 (***) were significant.

## Results

### Assessment of NCAPG2 Expression in Different Cancers and Normal Tissues

**Figure 1:**
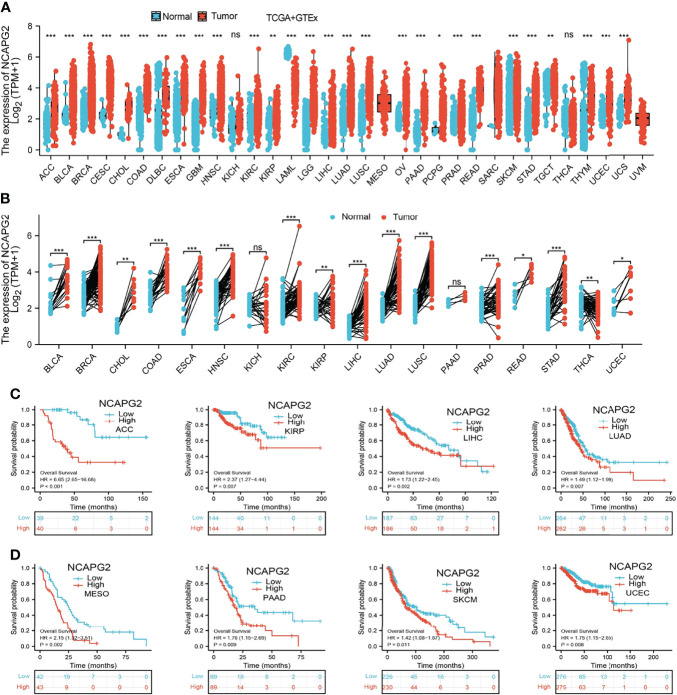
Expression pattern and prognostic value of NCAPG2 from the perspective of pan-cancer. **(A, B)** NCAPG2 expression levels in different tumor tissues and adjacent normal tissues from TCGA and GTEx databases. **(C, D)** Prognostic analysis of NCAPG2 mRNA expression levels in various human cancers. NS, *p* > 0.05, *p < 0.05, **p < 0.01, ***p < 0.001.

We explored the mRNA expression levels of NCAPG2 in tumor tissues and adjacent tissues from 33 types of cancer *via* the TCGA dataset and GTEX databases. Our research presented that the mRNA expression levels of NCAPG2 were increased in 28 of the 33 cancers compared with normal tissue (**Figure 1A**). We also examined NCAPG2 expression in paired cancer tissues and adjacent normal tissues in human cancer using the TCGA datasets. We found that NCAPG2 expression was significantly higher in 16 of the 18 cancers compared with normal tissue (**Figure 1B**). Furthermore, KM survival analysis and log-rank test were performed to assess the relationship between the mRNA expression levels of NCAPG2 and the OS of patients in pan-tumors and adjacent normal tissue types in the TCGA database. The results showed that higher NCAPG2 expression levels had a significant relationship with worse prognosis in ACC (adrenocortical carcinoma), KIRP (kidney renal clear cell carcinoma), LIHC (liver hepatocellular carcinoma), LUAD (lung adenocarcinoma), MESO (mesothelioma), PAAD (pancreatic adenocarcinoma), SKCM (skin cutaneous melanoma), and UCEC (uterine corpus endometrial carcinoma) (**Figures 1C, D**). Taking into account all the above analyses, and the combination of expression analysis and prognosis analysis, our research demonstrated the expression and prognostic value of NCAPG2 in pan-cancers.

### NCAPG2 Was Upregulated in Lung Adenocarcinoma

**Figure 2:**
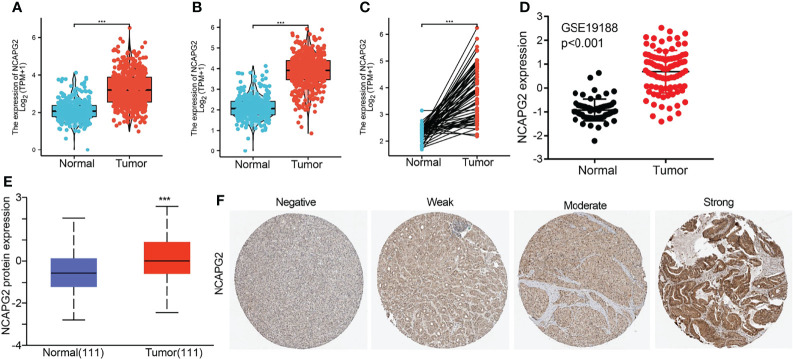
NCAPG2 RNA and protein expression in LUAD. **(A–C)** NCAPG2 mRNA expression levels in lung cancer patients and matched adjacent normal samples. **(D)** The expression level of NCAPG2 in lung cancer was determined by the GEO dataset. **(E, F)** NCAPG2 protein expression level based on CPTAC and Human Protein Atlas. ***p < 0.001.

We found that NCAPG2 expression was highly expressed in LUAD and LUSC (**Figures 2A, B**). We also confirmed that NCAPG2 expression was significantly higher in LUAD than in paired adjacent normal tissues (**Figure 2C**). To validate that, we used GEO datasets to assess the NCAPG2 expression in lung cancer. Results confirmed that NCAPG2 was significantly increased in lung cancer tissues compared to normal lung tissues (**Figure 2D**). UALCAN database results and immunohistochemistry (IHC) staining also suggested that the protein of NCAPG2 was upregulated in lung cancer tissues (**Figures 2E, F**). Taken together, NCAPG2 was upregulated in LUAD and might act as a pivotal player in the carcinogenesis of lung cancer.

### Overexpression of NCAPG2 Was Associated With Adverse Clinical Parameters in Lung Adenocarcinoma

**Figure 3:**
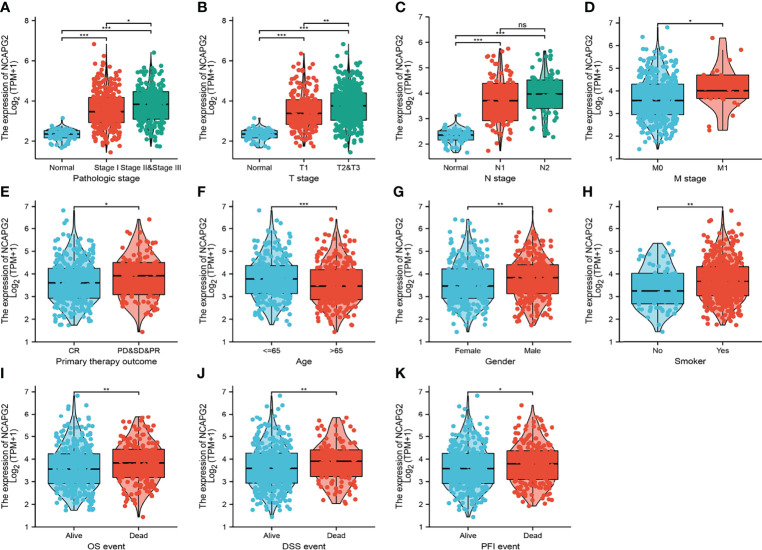
Clinical significance of NCAPG2 in lung adenocarcinoma. Correlation between NCAPG2 expression and clinical parameters, including **(A)** pathological stage, **(B–D)** TNM stage, **(E)** primary therapy outcomes, **(F)** age, **(G)** gender, **(H)** smoker, **(I–K)** OS event, DSS event, and PFS event. Primary therapy outcome: PD, progressive disease; SD, stable disease; PR, partial response; CR, complete response. NS, *p* > 0.05, *p < 0.05, **p < 0.01, ***p < 0.001.

We analyzed the relationship between NCAPG2 expression and diverse clinical features, including the pathological stage, TNM stage, primary therapy outcomes, age, gender, smoker, OS event, disease-specific survival (DSS) event, and progression-free survival (PFS) event. As shown in **Figures 3A–K**, an increase in NCAPG2 expression was significantly correlated with pathological stage, TNM stage, primary therapy outcomes, age, gender, smoker, OS event, DSS event, and PFS event (**Figures 3A–K** and **Table 1**). Logistic regression analysis results also suggested that the expression of NCAPG2 was significantly correlated with adverse clinicopathological prognosis, including T stage (T2, T3, and T4 vs. T1), N stage (N1, N2, and N3 vs. N0), M stage (M1 vs. M0), pathologic stage (Stage III and Stage IV vs. Stage I and Stage II), age (>65 vs. ≤65), and gender (male vs. female) (**Table 2**). These results suggested that a high NCAPG2 expression might positively correlate with the malignant phenotypes of LUAD.

**Table 1:** Clinical characteristics of the LUAD patients.

Characteristic	Low expression of NCAPG2	High expression of NCAPG2	*p*
*n*	267	268	
T stage, *n* (%)			0.003
T1	104 (19.5%)	71 (13.3%)	
T2	125 (23.5%)	164 (30.8%)	
T3	29 (5.5%)	20 (3.8%)	
T4	8 (1.5%)	11 (2.1%)	
N stage, *n* (%)			0.003
N0	187 (36%)	161 (31%)	
N1	45 (8.7%)	50 (9.6%)	
N2	24 (4.6%)	50 (9.6%)	
N3	0 (0%)	2 (0.4%)	
M stage, *n* (%)			0.004
M0	187 (48.4%)	174 (45.1%)	
M1	5 (1.3%)	20 (5.2%)	
Pathologic stage, *n* (%)			<0.001
Stage I	166 (31.5%)	128 (24.3%)	
Stage II	59 (11.2%)	64 (12.1%)	
Stage III	31 (5.9%)	53 (10.1%)	
Stage IV	6 (1.1%)	20 (3.8%)	
Gender, *n* (%)			0.002
Female	161 (30.1%)	125 (23.4%)	
Male	106 (19.8%)	143 (26.7%)	
Age, *n* (%)			0.006
≤65	112 (21.7%)	143 (27.7%)	
>65	147 (28.5%)	114 (22.1%)	
Smoker, *n* (%)			0.104
No	44 (8.4%)	31 (6%)	
Yes	213 (40.9%)	233 (44.7%)	
Age, median (IQR)	67 (60, 74)	63 (58, 71)	0.003

**Table 2:** Logistic regression analysis of the correlation between NCAPG2 expression and clinical pathological characteristics.

Characteristics	Total (*N*)	Odds Ratio (OR)	*p*-value
T stage (T2, T3, and T4 vs. T1)	532	1.763 (1.224–2.551)	0.002
N stage (N1, N2, and N3 vs. N0)	519	1.717 (1.187–2.495)	0.004
M stage (M1 vs. M0)	386	4.299 (1.699–13.141)	0.004
Pathologic stage (Stage III and Stage IV vs. Stage I and Stage II)	527	2.312 (1.498–3.619)	<0.001
Age (>65 vs. ≤65)	516	0.607 (0.428–0.859)	0.005
Gender (Male vs. Female)	535	1.738 (1.234–2.453)	0.002
Smoker (Yes vs. No)	521	1.553 (0.949–2.567)	0.082

### Diagnostic and Prognostic Value

**Figure 4:**
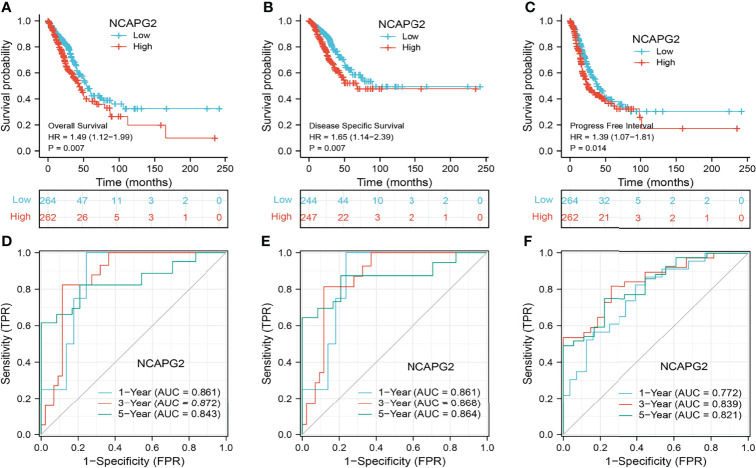
Prognostic and diagnostic value of NCAPG2 **(A–C)** Kaplan–Meier survival curves showed that lung adenocarcinoma patients with high NCAPG2 expression exhibited poor overall survival, disease-specific survival, and progression-free survival of NCAPG2 in LUAD determined by the TCGA-LUAD dataset. **(D–F)** Time-dependent ROC curves were used to determine the diagnostic value of NCAPG2 in lung adenocarcinoma.

**Figure 5:**
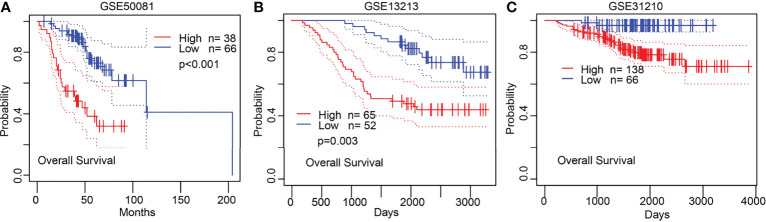
Validation of the prognosis of NCAPG2 in lung cancer. **(A–C)** Validation of the prognosis of NCAPG2 in lung cancer by GEO dataset.

KM survival analysis and log-rank test were performed to assess the relationship between the mRNA expression levels of NCAPG2 and the prognosis (OS, DSS, and PFS) of patients with LUAD in the TCGA database. The results showed that higher NCAPG2 expression was correlated with adverse OS, DSS, and PFS (**Figures 4A–C**). According to time-dependent ROC, the NCAPG2 expression level had a relatively good performance in predicting 1-year (C statistics, 0.861), 3-year (C statistics, 0.872), and 5-year OS (C statistics, 0.843) in LUAD patients (**Figure 4D**); had a better performance in predicting 1-year (C statistics, 0.861), 3-year (C statistics, 0.868), and 5-year disease-free survival (C statistics, 0.864) in LUAD patients (**Figure 4E**); and had a relatively good performance in predicting 1-year (C statistics, 0.772), 3-year (C statistics, 0.839), and 5-year PFS (C statistics, 0.821) in LUAD patients (**Figure 4F**). To validate the prognosis of NCAPG2 in lung cancer, we analyzed the GEO dataset and found that upregulation of NCAPG2 was correlated with poor prognosis in patients with lung cancer (**Figures 5A–C**).

### Predictive Value of the NCAPG2 Level Based on Clinical Subgroups

**Figure 6:**
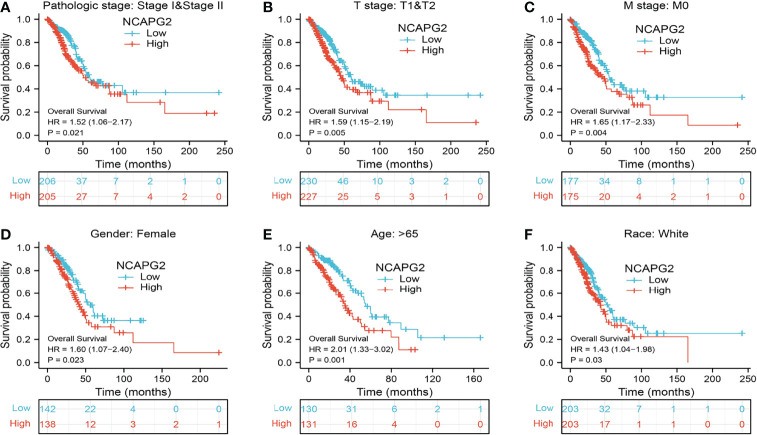
Associations between NCAPG2 expression level and the overall survival in different clinical subgroups of LUAD in the TCGA database. **(A)** Stage I–II, **(B)** T1–T2, **(C)** M0, **(D)** female, **(E)** age > 65, and **(F)** race, white.

To validate the prognosis of NCAPG2 in lung cancer, we subsequently determined the relationship between NCAPG2 expression and OS across different subgroups by various clinical features. The results consistently suggested that LUAD patients with higher NCAPG2 expression had a significantly deteriorative OS compared to those with a low NCAPG2 level, including the subgroup of stage I–II, T1–T2, M0, female, age>65, and race: white (**Figures 6A–F**).

### Gene Function Annotation and Pathway Analysis

**Figure 7:**
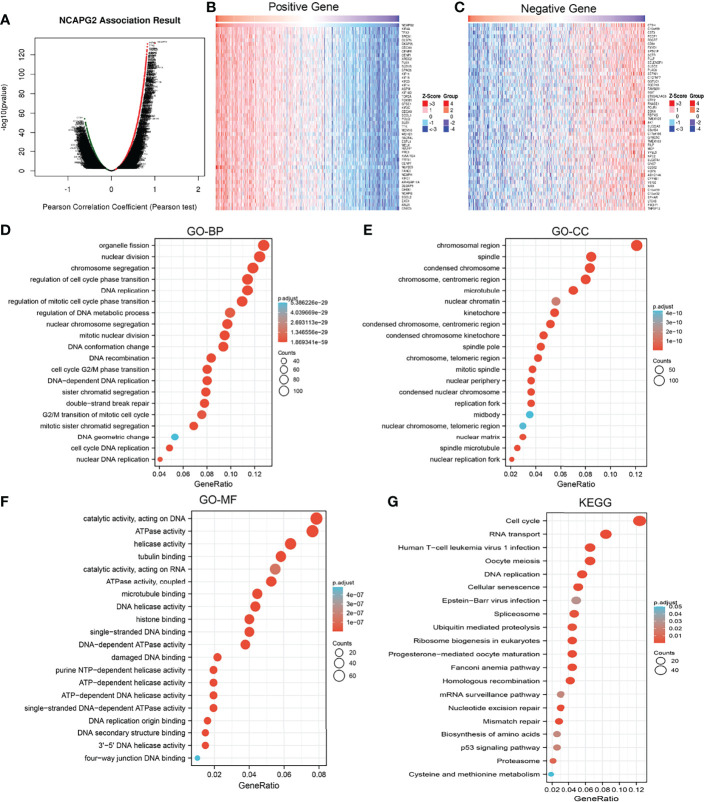
Functional enrichment analysis of NCAPG2 in LUAD. **(A–C)** The correlation analysis of NCAPG2 expression and its top 100 co-expressed gene network. **(D–G)** GO and KEGG enrichment analysis of co-expressed genes.

We used LinkedOmics to obtain the significant positive correlation with the NCAPG2 gene, and the heatmap shows 50 gene sets that were significantly positively or negatively correlated with NCAPG2n (**Figures 7A–C**). We conducted GO and KEGG analyses to perform functional annotations with NCAPG2. Functional annotations demonstrated that these genes were involved in organelle fission, nuclear division, chromosome segregation, regulation of cell cycle phase transition, DNA replication, and regulation of mitotic cell cycle phase transition (**Figure 7D**). Changes in the biological process (BP) and molecular function (MF) of NCAPG2 were correlated with chromosomal region, spindle, condensed chromosome, chromosome, centromeric region, microtubulenuclear chromatin, ATPase activity, helicase activity, tubulin binding, catalytic activity, acting on RNA, and ATPase activity (**Figures 7E, F**). KEGG molecular pathways were cell cycle, RNA transport, DNA replication, cellular senescence, spliceosome, ubiquitin-mediated proteolysis, and mRNA surveillance pathway (**Figure 7G**).

### NCAPG2-Related Signaling Pathways Based on Gene Set Enrichment Analysis

**Figure 8:**
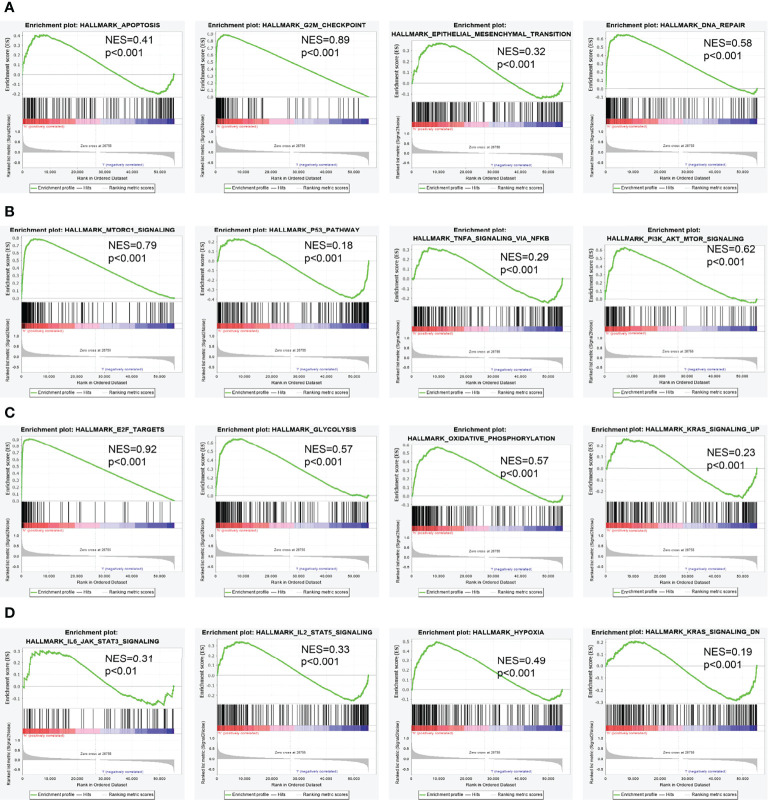
Identification of NCAPG2-related signaling pathways in lung adenocarcinoma. **(A–D)** Identification of NCAPG2-related signaling pathways by GSEA software.

To determine the biological function of NCAPG2, we analyzed the DEGs between the low and high NCAPG2 expression groups based on the median expression value of NCAPG2. Gene set enrichment analysis (GSEA) pathway analysis results confirmed that NCAPG2 is mainly involved in apoptosis, G2M checkpoint, epithelial–mesenchymal transition (EMT), DNA repair, mTORC1 signaling pathway, p53 signaling pathway, TNFα signaling pathway, PI3K-AKT-MTOR signaling pathway, IL-2 STAT5 signaling pathway, KRAS signaling pathway, hypoxia, glycolysis, oxidative phosphorylation, and IL-6 STAT3 signaling pathway (**Figures 8A–D**).

### Correlation Between NCAPG2 Expression and Immune Infiltration

**Figure 9:**
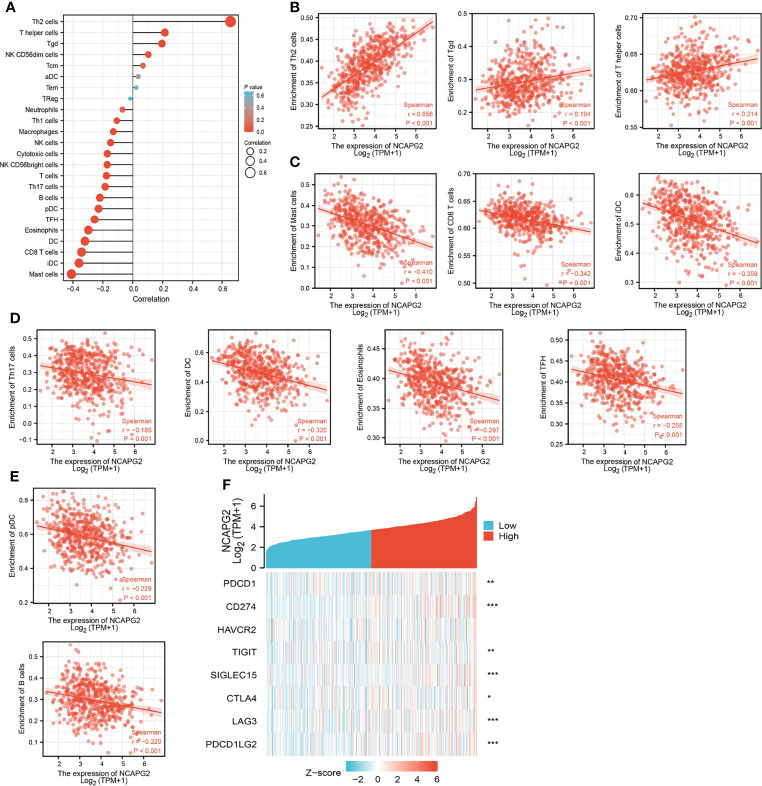
Correlation analysis of NCAPG2 expression and infiltration levels of immune cells in LUAD. **(A–E)** The correlation between NCAPG2 expression and infiltration levels of immune cells. **(F)** Correlation analysis of NCAPG2 expression and immune checkpoint-related genes in LUAD in the TCGA database.

Tumor-infiltrated lymphocyte cells play a key role in cancer progression and affect the prognosis of lung cancer patients. Therefore, we next examine whether NCAPG2 is correlated with the immune infiltration level in LUAD. Our finding suggested that mRNA expression levels of NCAPG2 had a significant positive association with Th2 cells, T helper cells, and Tgd. On the contrary, NCAPG2 expression was negatively correlated with T cells, Th17 cells, B cells, pDC, TFH, eosinophils, DC, CD8 T cells, iDC, and mast cells (**Figures 9A–E**).

### The Relationship Between NCAPG2 and Immune Checkpoints in LUAD

Considering that NCAPG2 might be the potential oncogene in LUAD, the relationship of NCAPG2 with PDCD1, CD274, HAVCR2, TIGIT, SIGLEC15, CTLA4, LAG3, and PDCD1LG2 in LUAD was assessed. As a result, we found that the expression levels of NCAPG2 had a significant positive correlation with PDCD1 (PD-1), CD274 (PD-L1), TIGIT, SIGLEC15, CTLA4, LAG3, and PDCD1LG2 (PD-L2) in LUAD (**Figure 9F**). These results indicated that tumor immune escape and antitumor immunity might be involved in NCAPG2-mediated carcinogenic processes of LUAD.

### Prognostic Potential of NCAPG2 Expressions in LUAD Based on Immune Cells

**Figure 10:**
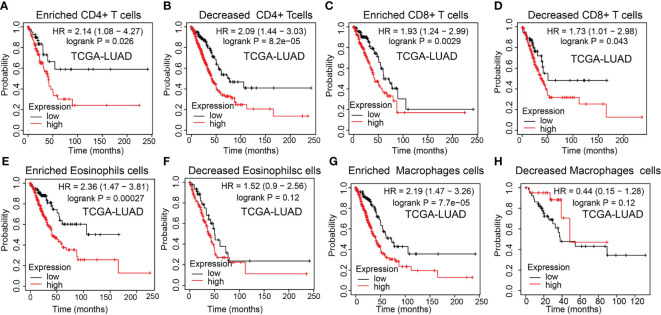
Kaplan–Meier survival curves according to the high and low expression of NCAPG2 in immune cell subgroups in LUAD. **(A–H)** Correlations between NCAPG2 expression and overall survival in different immune cell subgroups in LUAD patients were determined by Kaplan–Meier plotter.

The above results suggested that NCAPG2 was associated with the immune infiltration of LUAD. Also, increased NCAPG2 had a worse prognosis in LUAD patients. Thus, we proposed a hypothesis that NCAPG2 may affect the prognosis of LUAD patients partly through immune infiltration. Then, we performed KM plotter analysis of NCAPG2 expression in LUAD following CD4+ memory T cells, CD8+ T cells, macrophages, and eosinophilic cells. We found that higher NCAPG2 levels in LUAD in enriched CD4+ memory T cells, CD8+ T cells, macrophages, and eosinophilic cells had a worse prognosis (**Figures 10A–H**). The above results suggested that immune infiltration may, in part, affect the high NCAPG2 expression prognosis of LUAD patients.

### Depletion of *NCAPG2* Significantly Suppressed Proliferation, Migration, and Invasion of LUAD Cells

**Figure 11:**
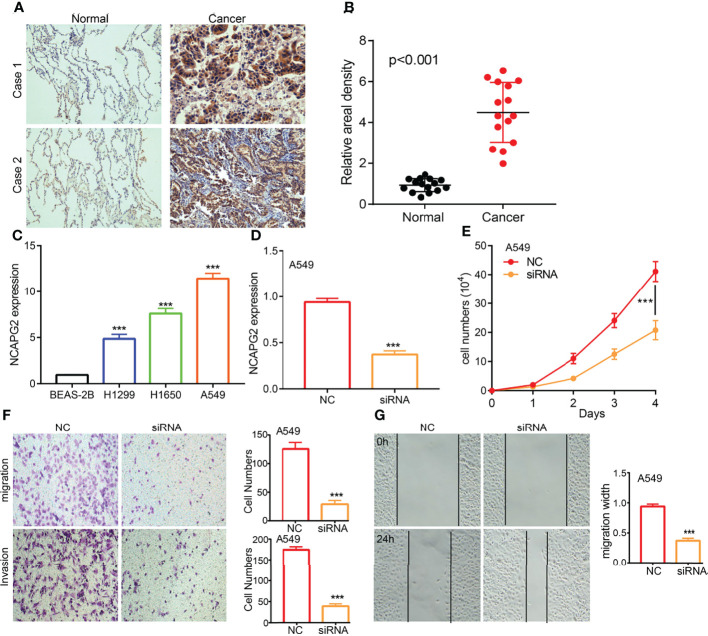
NCAPG2 regulates LUAD cell proliferation, cell migration, and cell invasion. **(A, B)** IHC assay detects the protein of NCAPG2 in lung adenocarcinoma cancerous cell lines. **(C)** qPCR assay examines the expression level of NCAPG2 in lung adenocarcinoma cancerous cell lines, including A549, H1299, and H1650, compared to the normal human bronchial epithelial cell line: BEAS-2B. **(D)** Validation the knock down efficiency of NCAPG2 in A549 cells by using qPCR assay. **(E–G)** Knockdown of NCAPG2 inhibited cell proliferation and migration examined by CCK8 and transwellassay.NC, negative control; siRNA, NCAPG2 siRNA; ***p < 0.001.

To examine the expression of *NCAPG2*, we detected *NCAPG2* expression levels in LUAD tissues and cell lines using IHC and qRT-PCR assay. Results confirmed that *NCAPG2* was significantly increased in lung cancer tissues and cell lines, especially in A549 and H1650 cells (**Figures 11A–C**). The qRT-PCR assay showed that the expression of *NCAPG2* mRNA was significantly decreased in A549 cells after treatment with targeted siRNA (**Figure 11D**). The growth curve and transwell assays demonstrated that NCAPG2 depletion significantly inhibited the cell proliferation, cell migration, and cell invasion abilities of LUAD (**Figures 11E–G**). Collectively, these results demonstrated that *NCAPG2* was highly expressed in LUAD and significantly affected their proliferation, cell migration, and cell invasion.

### Analysis of Upstream miRNAs of NCAPG2

**Figure 12:**
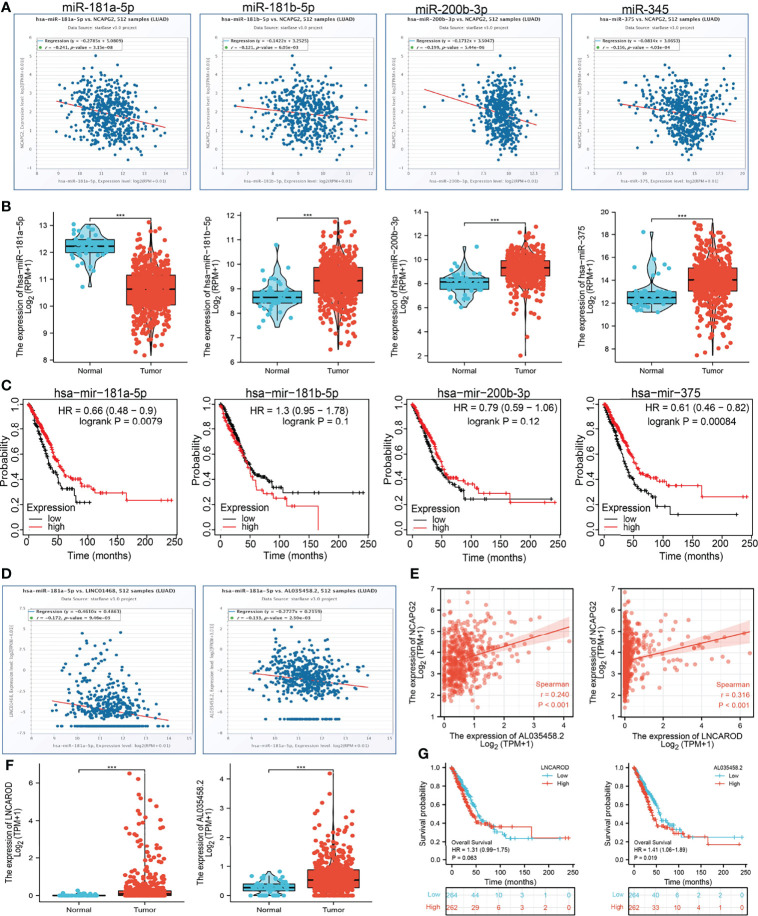
LncRNA/hsa-miR-181a-5p/NCAPG2 regulatory network. **(A)** Analysis of the correlations between NCAPG2 expression and hsa-miR-181a-5p, hsa-miR-181b-5p, hsa-miR-200b-3p, and hsa-miR-345 in TCGA-LUAD. **(B)** Analysis of hsa-miR-181a-5p, hsa-miR-181b-5p, hsa-miR-200b-3p, and hsa-miR-345 expression in lung cancer and adjacent normal tissues in the TCGA database. **(C)** Association between hsa-miR-181a-5p, hsa-miR-181b-5p, hsa-miR-200b-3p, and hsa-miR-345 expression and LUAD patients’ outcomes. **(D)** Correlations between hsa-miR-181a-5p expression and AL035458.2 and LINC01468 in LUAD. **(E)** Correlations between NCAPG2 expression and AL035458.2 and LINC01468 in LUAD. **(F)** Analysis of AL035458.2 and LINC01468 expression in lung cancer and adjacent normal tissues in the TCGA database. **(G)** Association between AL035458.2 and LINC01468 expression and LUAD patients’ outcomes. ***p < 0.001.

Increasing evidence confirmed that ncRNAs regulate gene expression in various manners at almost every step (19, 20). To unravel whether NCAPG2 was regulated by some ncRNAs, we predicted the upstream miRNAs that might bind to NCAPG2, and finally found 89 miRNAs (**Supplementary Table 1**). There should be a negative relationship between NCAPG2 and upstream miRNA because of the mechanism by which upstream miRNAs negatively regulated the expression of NCAPG2 at the post-transcriptional level. Thus, the correlation between NCAPG2 and 89 miRNAs was detected in the TCGA-LUAD database. As a result, our study showed that the expression levels of NCAPG2 were significantly negatively associated with hsa-miR-181a-5p, hsa-miR-181b-5p, hsa-miR-200b-3p, and hsa-miR-345, and that there were no statistical expression relationships between NCAPG2 and other miRNAs in LUAD (**Figure 12A**). Then, we determined the RNA expression levels of hsa-miR-181a-5p, hsa-miR-181b-5p, hsa-miR-200b-3p, and hsa-miR-345 in the TCGA-LUAD database. The results demonstrated that hsa-miR-181a-5p expression levels in LUAD were lower than in adjacent normal tissue control (**Figure 12B**). We also explored the correlation between the expression levels of hsa-miR-181a-5p, hsa-miR-181b-5p, hsa-miR-200b-3p, and hsa-miR-345 and LUAD patient prognosis in the TCGA database. Our research showed that the higher expression of hsa-miR-181a-5p was significantly associated with good outcomes in LUAD (**Figure 12C**). With the combination of correlation analysis, expression analysis, and survival analysis, we suggested that hsa-miR-181a-5p might be the most likely regulatory miRNA of NCAPG2 in LUAD.

### Analysis of Upstream Potential lncRNAs of hsa-miR-181a-5p

We used the ENCORI database to predict upstream potential lncRNAs that interact with hsa-miR-181a-5p. Finally, we selected 143 candidate lncRNAs that interact with hsa-miR-181a-5p (**Supplementary Table 2**). The competitive endogenous RNA (ceRNA) hypothesis suggests that lncRNA competitively binds tumor-suppressive miRNAs to reduce the suppressive miRNA effect on target mRNAs. Thus, there should be a negative correlation between lncRNA and target miRNA, while there is a positive correlation between lncRNA and target mRNA in the ceRNA network. Therefore, we assessed expression correlations of the miR-181a-5p/NCAPG2 and 143 lncRNAs in the TCGA-LUAD database. The results highlighted that only AL035458.2 and LINC01468 (LncAROD) were positively associated with NCAPG2 and negatively associated with hsa-miR-181a-5p (**Figures 12D, E**). We next performed AL035458.2 and LINC01468 expression analysis in LUAD *via* the TCGA set. The results showed that AL035458.2 and LINC01468 expression levels were significantly upregulated in LUAD compared with normal controls (**Figure 12F**). Subsequently, we detected the prognostic values of AL035458.2 and LINC01468 in LUAD by using the TCGA database. The result demonstrated that higher expression levels of AL035458.2 were significantly associated with worse outcome in LUAD. However, we found that the expression levels of LINC01468 had no correlation with prognosis in LUAD patients (**Figure 12G**). Considering correlation analysis, expression analysis, and survival analysis, we chose AL035458.2 as the most likely upstream lncRNA of the NCAPG2/hsa-miR-181a-5p axis in LUAD.

## Discussion

In this study, we examined the mRNA expression levels of NCAPG2 in pan-tumors and corresponding adjacent normal tissues using the TCGA and GTEX databases. Taking our expression analysis and validation analyses together, NCAPG2 was upregulated in various human cancers. Meanwhile, we found that NCAPG2 RNA and protein expression were increased in LUAD tissues and correlated with adverse clinical features, including pathological stage, TNM stage, and OS event. ROC curve analysis indicated that NCAPG2 may be a promising diagnostic biomarker for differentiating LUAD from normal tissues. KM curves suggested that NCAPG2 expression is correlated with OS, disease-free survival, and PFS in LUAD patients of the TCGA and GEO datasets. Our IHC assay also demonstrated that NCAPG2 was overexpressed in lung cancer. These results show that NCAPG2 plays an important role in the progression of lung cancer. Our findings are consistent with previous research. NCAPG2 was elevated in diverse human cancer tissues and correlated with clinicopathological features and poor prognosis (6, 7).

Previous studies reported that NCAPG2 is necessary for kinetochore localization and cell cycle (21). In this study, we investigated the underlying mechanisms through which NCAPG2 affected the progression of LUAD. GSEA enrichment confirmed that NCAPG2 was significantly associated with apoptosis, G2M checkpoint, EMT, DNA repair, mTORC1 signaling pathway, p53 signaling pathway, TNFα signaling pathway, PI3K-AKT-MTOR signaling pathway, IL-2 STAT5 signaling pathway, KRAS signaling pathway, hypoxia, glycolysis, oxidative phosphorylation, and IL-6 STAT3 signaling pathway.

It has been shown that NCAPG was highly expressed in NSCLC and associated with adverse prognosis and immune infiltration (22). In this study, we found that NCAPG2 expression had a significant positive association with Th2 cells, T helper cells, and Tgd. On the contrary, NCAPG2 expression was negatively correlated with T cells, Th17 cells, B cells, pDC, TFH, eosinophils, DC, CD8 T cells, iDC, and mast cells. Moreover, we confirmed that the expression levels of NCAPG2 had a significant positive correlation with PDCD1 (PD-1), CD274 (PD-L1), TIGIT, SIGLEC15, CTLA4, LAG3, and PDCD1LG2 (PD-L2) in LUAD. These results indicated that tumor immune escape and antitumor immunity might be involved in NCAPG2-mediated carcinogenic processes of LUAD. More importantly, we found that NCAPG2 may affect the prognosis of LUAD patients partly through immune infiltration.

Given that GSEA enrichment results show that NCAPG2 may play a central role in cell proliferation and cell migration, we decided to examine the potential biological function of NCAPG2 in LUAD. *In vitro*, we found that NCAPG2 was upregulated in LUAD tissues and cell lines. Knockdown of NCAPG2 in A549 cells inhibited cell proliferation, migration, and cell invasion in LUAD. Based on the above finding, we proposed that NCAPG2 exerts an essential function in regulating the pathologic progression of LUAD.

To further uncover the potential upregulation of NCAPG2 in LUAD, we conducted a correlation analysis, an expression analysis, and a survival analysis of these miRNAs in LUAD. Our study showed that NCAPG2 was significantly negatively associated with hsa-miR-181a-5p. Hsa-miR-181a-5p expression levels were lower than in adjacent normal tissue control in LUAD. Survival analysis showed that the higher expression of hsa-miR-181a-5p was significantly associated with good outcomes in LUAD. The ceRNA hypothesis suggested that there should be a negative relationship between NCAPG2 and upstream miRNA because of the mechanism by which upstream miRNAs negatively regulated the expression of NCAPG2 at the post-transcriptional level. Combining the ceRNA hypothesis, correlation analysis, expression analysis, and survival analysis, we suggested that hsa-miR-181a-5p might serve as the most likely regulatory miRNA of NCAPG2 in LUAD. Our research demonstrated that hsa-miR-181a-5p might be a negative regulator of pancreatic cancer *via* target NCAPG2.

We selected 143 upstream potential lncRNAs that interacted with hsa-miR-181a-5p by using the ENCORI database. Based on the ceRNA hypothesis, there should be a positive correlation between potential lncRNA and NCAPG2 and a negative correlation between potential lncRNA and hsa-miR-181a-5p, and it should be oncogenic lncRNA in LUAD. By correlation analysis, survival analysis, and expression analysis, AL035458.2 was selected as the most likely upstream lncRNA of the NCAPG2/hsa-miR-181a-5p axis in LUAD. AL035458.2’s Ensembl ID is ENSG00000250917. AL035458.2 is a novel lncRNA transcript. There are only a few studies on AL035458.2, and thus, it is important to be studied further. Taken together, the AL035458.2/hsa-miR-181a-5p/NCAPG2 axis was well identified as a potential regulatory pathway in LUAD.

This study improves our understanding of the correlation between NCAPG2 and LUAD, but some limitations still exist. First, although we explored the correlation between NCAPG2 and immune infiltration in LUAD patients, there is a lack of experiments to validate the function of NCAPG2 in the tumor microenvironment regulation of LUAD. Second, we reveal that knockdown of NCAPG2 inhibits cell proliferation, cell migration, and cell invasion of LUAD. However, the potential molecular mechanisms of NCAPG2 in cancer progression need to be explored in further studies.

## Conclusion

This study describes, for the first time, the clinical relevance, immuno-oncology features, and biological function of NCAPG2 in LUAD. In summary, NCAPG2 is a promising prognostic factor, and its future application may help determine the optimal treatment strategy for lung adenocarcinoma.

## Data Availability Statement

The original contributions presented in the study are included in the article/**Supplementary Material**. Further inquiries can be directed to the corresponding authors.

## Author Contributions

XC, JG, and WR designed this work and performed the related assay. FZ analyzed the data. XN and XJ supervised and wrote the manuscript. All authors have read and approved the final version of the manuscript.

## Funding

This study was supported by the Applied Basic Research Project of Yunnan Provincial Science and Technology Department and Kunming Medical University, No. 2020001AY070001-117, and the Open Project of The First People’s Hospital of Yunnan Province Clinical Medicine Center (2021LCZXXF‐XZ03).

## Conflict of Interest

The authors declare that the research was conducted in the absence of any commercial or financial relationships that could be construed as a potential conflict of interest.

## Publisher’s Note

All claims expressed in this article are solely those of the authors and do not necessarily represent those of their affiliated organizations, or those of the publisher, the editors and the reviewers. Any product that may be evaluated in this article, or claim that may be made by its manufacturer, is not guaranteed or endorsed by the publisher.
