# Supplementary material for: LncRNA-AL035458.2/hsa-miR-181a-5p Axis-Mediated High Expression of NCAPG2 Correlates With Tumor Immune Infiltration and Non-Small Cell Lung Cancer Progression
Source: Front Oncol. 2022 May 19;12:910437. doi: 10.3389/fonc.2022.910437 (PMC9160743; doi:10.3389/fonc.2022.910437)
Supplement: Supplementary file 1 [file Table_1.docx]

**Supplementary Table 1 The potential upstream miRNAs of NCAPG2**

| hsa-miR-17-5p | hsa-miR-656-3p | hsa-miR-410-3p | |
| --- | --- | --- | --- |
| hsa-miR-20a-5p | hsa-miR-361-3p | hsa-miR-376b-3p | |
| hsa-miR-24-3p | hsa-miR-340-5p | hsa-miR-485-3p | |
| hsa-miR-93-5p | hsa-miR-340-5p | hsa-miR-432-5p | |
| hsa-miR-106a-5p | hsa-miR-488-3p | hsa-miR-495-3p | |
| hsa-miR-192-5p | hsa-miR-501-3p | hsa-miR-520e | |
| hsa-miR-7-5p | hsa-miR-502-3p | hsa-miR-520a-5p | |
| hsa-miR-181a-5p | hsa-miR-556-3p | hsa-miR-525-5p | |
| hsa-miR-181b-5p | hsa-miR-616-3p | hsa-miR-520b | |
| hsa-miR-181c-5p | hsa-miR-624-3p | hsa-miR-520c-3p | |
| hsa-miR-181d-5p | hsa-miR-654-3p | hsa-miR-520d-3p | |
| hsa-miR-200b-3p | hsa-miR-300 | hsa-miR-516b-5p | |
| hsa-miR-140-5p | hsa-miR-875-5p | hsa-miR-513a-5p | |
| hsa-miR-126-5p | hsa-miR-665 | hsa-miR-532-5p | |
| hsa-miR-375 | hsa-miR-873-5p | hsa-miR-579-3p | |
| hsa-miR-186-5p | hsa-miR-543 | hsa-miR-584-5p | |
| hsa-miR-200c-3p | hsa-miR-374b-5p | hsa-miR-641 | |
| hsa-miR-106b-5p | hsa-miR-513c-5p | hsa-miR-4677-3p | |
| hsa-miR-302a-3p | hsa-miR-1301-3p | hsa-miR-4703-5p | |
| hsa-miR-365a-3p | hsa-miR-1270 | hsa-miR-4766-5p | |
| hsa-miR-302b-3p | hsa-miR-302e | hsa-miR-4766-3p | |
| hsa-miR-302c-3p | hsa-miR-675-3p | hsa-miR-5047 | |
| hsa-miR-302d-3p | hsa-miR-2681-3p | hsa-miR-5688 | |
| hsa-miR-369-3p | hsa-miR-2682-5p | hsa-miR-873-3p | |
| hsa-miR-373-3p | hsa-miR-3146 | hsa-miR-1277-5p | |
| hsa-miR-374a-5p | hsa-miR-3163 | hsa-miR-3690 | |
| hsa-miR-376a-3p | hsa-miR-514b-5p | hsa-miR-3942-5p | |
| hsa-miR-381-3p | hsa-miR-3200-5p | hsa-miR-4428 | |
| hsa-miR-323a-3p | hsa-miR-3617-5p | hsa-miR-346 | |
| hsa-miR-429 | hsa-miR-20b-5p |  |  |

**Supplementary Table 2 The potential upstream lncRNAs of miR-181a-5p**

| LINC01355 | LINC00847 | AC124312.3 | ADIRF-AS1 | |
| --- | --- | --- | --- | --- |
| SNHG12 | AC008443.1 | SNHG14 | LINC01514 | |
| AL513327.3 | AL137003.2 | OIP5-AS1 | KCNQ1OT1 |  |
| SLFNL1-AS1 | HCG11 | AC022306.2 | LINC00294 | |
| AC239868.1 | ZSCAN16-AS1 | GABPB1-IT1 | AP001350.2 | |
| AL390728.6 | AL049543.1 | AC011912.1 | SNHG1 |  |
| AC098828.2 | Z97832.2 | AC116913.1 | NEAT1 |  |
| AL133243.4 | SNHG5 | AC068338.2 | MALAT1 |  |
| AC007744.1 | SYNJ2-IT1 | AC105339.2 | AP002761.4 | |
| ALMS1-IT1 | PSMG3-AS1 | LINC01579 | AP002884.1 | |
| AC079117.1 | AC005154.1 | AL031009.1 | MIR4697HG | |
| PAX8-AS1 | ST7-AS1 | CRNDE | AC092747.4 | |
| LINC01806 | AC000123.3 | AC092127.1 | AC008014.1 | |
| AC009962.1 | AC010655.4 | FENDRR | AC008147.2 | |
| AC012513.3 | SNHG6 | AC009113.1 | AC126474.2 | |
| THUMPD3-AS1 | AC009902.3 | AC126365.1 | HELLPAR |  |
| AC112220.2 | CASC19 | AC233702.9 | AC144548.1 | |
| AC124045.1 | PVT1 | AC005562.1 | AL161772.1 | |
| AC112512.1 | ERICD | AC127024.5 | N4BP2L2-IT2 | |
| AC135507.1 | AL158206.1 | LINC00910 | STARD13-IT1 | |
| PSMD6-AS2 | CDKN2B-AS1 | RUNDC3A-AS1 | INTS6-AS1 | |
| MBNL1-AS1 | AL161729.3 | AC091152.4 | AL138955.1 | |
| AC080013.1 | AL138756.1 | AC004477.3 | LINC01232 | |
| AC010442.1 | SNHG7 | AC009720.1 | LINC00641 | |
| MIR4458HG | AL137186.1 | AC005332.6 | AL133453.1 | |
| AC091946.2 | ZEB1-AS1 | AC145207.5 | PSMA3-AS1 | |
| LIFR-AS1 | RP11-592B15.3 | AP005136.2 | AL163051.2 | |
| LUCAT1 | SGMS1-AS1 | LINC00667 | MEG3 |  |
| AC104109.4 | LINC01468 | AC006249.1 | MEG8 |  |
| AC021078.1 | AL132656.2 | AC087683.2 | SNHG14 |  |
| AC120349.2 | AC008555.8 | AL035458.2 | AC012313.1 | |
| AC008543.1 | LINC00665 | NORAD | MIAT |  |
| AC020917.4 | AC021092.1 | AL031686.1 | AL022322.2 | |
| AC008736.1 | IGFL2-AS1 | AL031666.3 | Z82186.1 |  |
| AC010624.2 | AC022150.4 | DSCAM-AS1 | ZNF674-AS1 | |
| GUSBP11 | XIST | DANT2 |  |  |
